# Ultra-broadband and compact graphene-on-silicon integrated waveguide mode filters

**DOI:** 10.1038/s41598-018-28076-8

**Published:** 2018-06-29

**Authors:** Peng Xing, Kelvin J. A. Ooi, Dawn T. H. Tan

**Affiliations:** Photonic Devices and Systems Group, Singapore University of Technology and Design, 8 Somapah Rd, 487372 Singapore, Singapore

## Abstract

Increasing bandwidth demands in optical communications necessitates the introduction of mode-division multiplexing (MDM) on top of the existing wavelength-division multiplexing (WDM) systems. Simultaneous management of both multiplexing systems will be a complex task, and there is the possibility of signal degradation through modal crosstalk. Here, we propose graphene-on-silicon (GOS) integrated waveguide mode filters to suppress the propagation of spurious waveguide modes at the telecommunications wavelength. Graphene’s high fabrication tolerance potentially enables surgical tailoring and deployment at targeted segments on the waveguide to absorb the undesired TE_0_ or TE_1_ modes. The proposed GOS waveguide mode filters can potentially improve the performance and reduce the device footprint of MDM systems.

## Introduction

The scaling of bandwidth in optical communication systems is largely enabled by the invention of wavelength-division multiplexing (WDM) technologies four decades ago^1^, and is instrumental to the large-scale adoption of optical fiber communications technology today. WDM’s contemporary was mode-division multiplexing (MDM), which was developed at the same epoch albeit with less success due to the issue of modal crosstalk that limits the propagation length^2^. Since then, the growth of bandwidth density in WDM systems has reached its limitations and thus cannot keep up with the ever-inflationary demands. As such, the implementation of MDM on photonic integrated circuits has recently regained research attention in attempts to supplement the diminishing returns of WDM systems^3–5^.

A myriad of MDM systems are currently being studied, ranging from the asymmetric directional coupler (ADC)^3–11^, Bragg gratings^4,12–15^, and the multimode interference (MMI) coupler^16–19^. ADCs usually suffer from large device footprint, with the coupling coefficient highly dependent on the waveguide dimensions as well as the operating wavelength. As such, MDM systems based on ADCs have limited spectrum bandwidth and are usually stringent in their fabrication tolerances. On the other hand, while Bragg gratings allow for the most compact device design, its bandwidth is limited by the Bragg period, and small fabrication imperfections would alter the photonic bandgap.

It is envisioned that when WDM and MDM systems are gradually implemented onto the same platform, the management of the propagating signals in the network becomes more complex, and signal degradation through modal crosstalk is highly probable. As such, there is a need for mode-filter components to suppress the propagation of spurious waveguide modes. Conventional photonic waveguide mode filters often rely on the effective index differences between the desired and unwanted modes, and filter the latter using optically resonant devices like Bragg gratings and photonic crystal waveguides^4,12–15^. These devices have downsides of very narrow spectral bandwidth, low fabrication tolerances, and the build-up of the reflected optical modes may be complex to manage.

Here, we propose graphene-on-silicon (GOS) waveguides as a straightforward technology to implement the waveguide mode filters. Graphene is a thin film material which strongly absorbs light at a very broad spectrum bandwidth^20^. Graphene has been deployed for the design of photonic devices such as broadband polarizers^21^, modulators^22–25^, detectors^26,27^, ultrafast lasers^28^, sensors^29^, plasmonics devices^30^, and nonlinear devices^31–33^. Recently, graphene has also been successfully embedded in a polymer substrate to demonstrate broadband spatial-mode filtering with high extinction ratios^34^. The ease of deployment of graphene films enables us to surgically place graphene layers at targeted segments on the waveguide surface that differentially absorb light of either the TE_0_ or TE_1_ modes. Owing to the unique optical properties of graphene, our proposed mode filters have relatively high selection and extinction ratios, broad spectrum bandwidths, low back reflection, and very good device fabrication tolerances, which will be discussed in detail.

## Design of the Graphene-on-Silicon Waveguide Mode Filter

### Structural design

**Figure 1:**
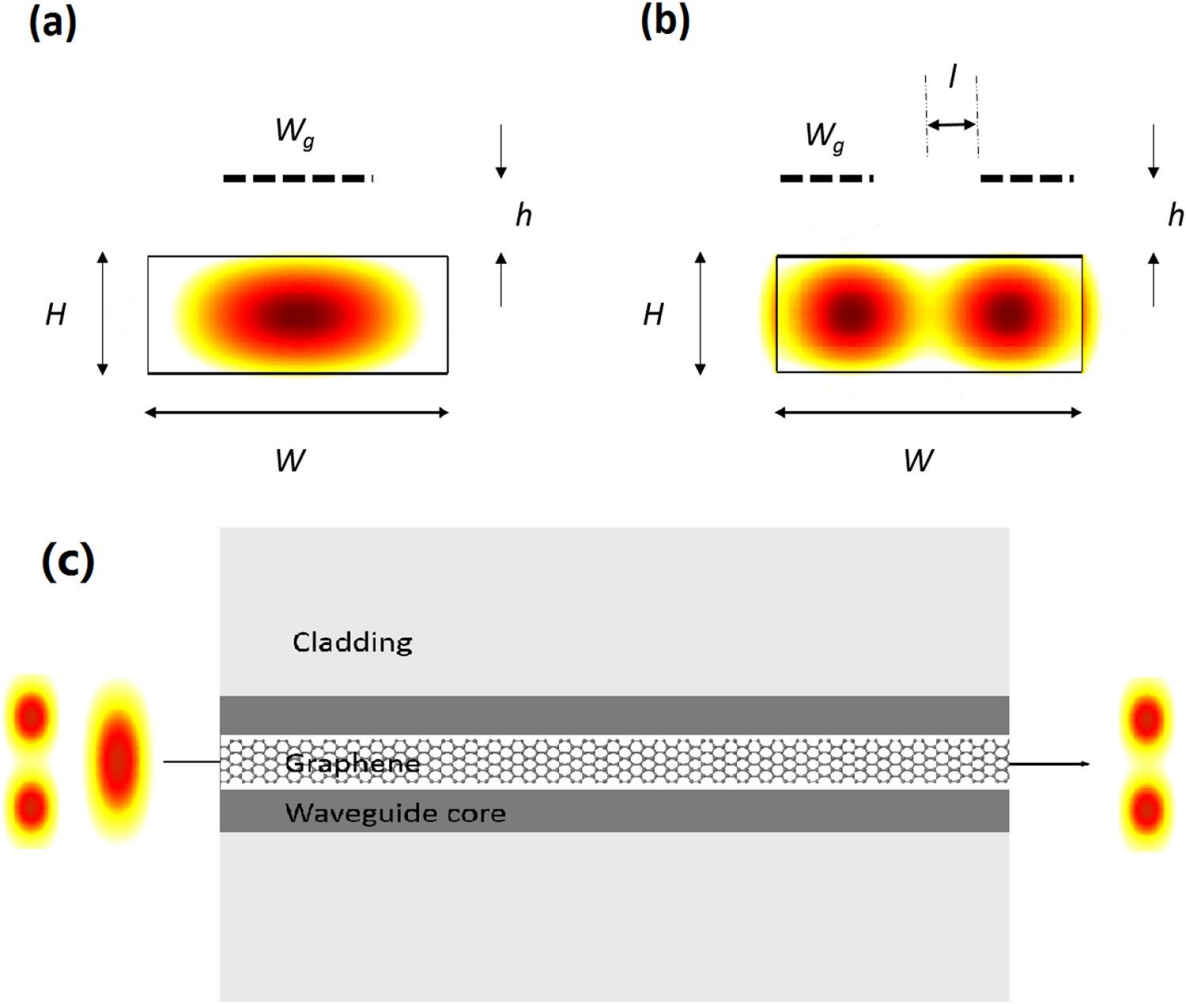
Configurations of the waveguide mode filters. Graphene’s dimensions are surgically tailored and strategically placed to absorb the specific undesired (**a**) TE_0_ or (**b**) TE_1_ optical waveguide modes. (**c**) Top view of TE_0_ filter.

Figure 1 shows the schematic design of two GOS waveguide mode filters for modes TE_0_ and TE_1_, respectively, at the wavelength of 1.55 µm. A close observation of the modal electric-field intensities reveals their different spatial configurations within the same waveguide cross-section. Figure 1(a) depicts the propagation of the TE_0_ mode which has only one lobe at the center of the waveguide, while Fig. 1(b) depicts the TE_1_ mode having two lobes side-by-side, with the peak electric-field intensities displaced from the center. Hence, it follows that the magnitude of the modal optical absorption can be tuned by varying the dimensions of the graphene layer, as well as strategically targeting segments of the waveguide where the peak intensities are located, as depicted by the dashed-lines in Fig. 1.

In these two GOS mode filters, the undesired modes will be absorbed by the graphene layer. Then the light will not be reflected back or scattered out the waveguides to cause signal noise in other photonic circuits. Since the graphene thickness is only 0.3 nm, the presence of graphene layer will highly increase the imaginary part of waveguide effective index. However, the graphene layer can only cause negligible change in the waveguide mode profile. Then the incident light will have negligible reflection at the input end of the mode filters.

There are two important design parameters used to characterize the performance of the waveguide mode filters. The first design parameter is the extinction ratio per unit length (*ER*) between the pass-mode (the allowed mode) and the stop-mode (the filtered mode), denoted by

1 $$ER={\alpha }_{stop}-{\alpha }_{pass}$$

where *α* is the effective waveguide absorption coefficient in units dB cm^−1^. The magnitude of *ER* will determine the compactness of the device, given that the effective length of the waveguide, *L*, and the intensity contrast, *C* (in units dB), is related by *L* = *C/ER*.

The second design parameter is the selection ratio (*SR*), defined as the ratio between *ER* and the absorption coefficient of the pass-mode, written as

2 $$SR=\frac{{\alpha }_{stop}-{\alpha }_{pass}}{{\alpha }_{pass}}$$

The SR determines how well the stop-mode can be filtered out with minimal reduction in the intensity of the pass-mode, i.e. quantifying the insertion loss (*IL*) of the device. Since *IL* = *α*_*pass*_ × *L*, we can easily relate the quantities *C* and *SR* through *IL* = *C/SR*. This shows that a high *SR* is preferred to keep the insertion losses low.

Finally, the main goal of the device design is to obtain the lowest *IL* and *L* possible. This can be evaluated from the *IL-L* product, given as

3 $$IL\times L=\frac{{C}^{2}}{ER\times SR}$$

From here, a good design guideline can be obtained from the *ER-SR* product metric. In general, we would design for a high *ER-SR* product to give a low *IL-L* product. Nonetheless, *ER* and *SR* for each device may still need to be assessed individually to meet specification requirements, for example, a stringent 3 dB insertion loss limit, or a minimum of 10 dB signal contrast requirement.

The GOS waveguide mode filters will be designed based on the parameters mentioned above. The TE_0_ filter in Fig. 1(a) designates TE_0_ as the stop-mode and TE_1_ as the pass-mode, and vice-versa for the TE_1_ filter in Fig. 1(b). The graphene layer(s) with width *W*_*g*_ will be placed on the targeted silicon waveguide surface segments, taking into account a possible small separation of *h* that arises from fabrication imperfections. For the TE_1_ filter, narrower graphene strips will be placed with a displacement, *l*, from the waveguide center. Finally, the waveguide dimensions are denoted as *W* (width) and *H* (height) respectively. A mode-analysis simulation would be conducted to study how *ER* and *SR* are affected by the interplay between these structural and geometrical dimensions.

### Graphene as an efficient optical absorber

Graphene is a material with a unique two-dimensional (2-D) electronic band structure that confers exceptional optical properties. The linear 2-D optical conductivity of graphene is well described by the Kubo formula, given in its local analytical form as^35^

4 $$\begin{array}{rcl}{\sigma }_{g}(\omega ) & = & \frac{i{e}^{2}(2{k}_{B}T)}{\pi {\hslash }^{2}(\omega +i{\nu }_{1})}\{\frac{{E}_{F}}{2{k}_{B}T}+\,\mathrm{ln}[2\exp (-\frac{|{E}_{F}|}{{k}_{B}T})+1]\}\\  &  & +\frac{{e}^{2}}{4\hslash }\{0.5+\frac{1}{\pi }{\tan }^{-1}[\frac{\hslash (\omega +i{\nu }_{2})-2|{E}_{F}|}{2{k}_{B}T}]\\  &  & -\frac{i}{2\pi }\,\mathrm{ln}(\frac{{[\hslash (\omega +i{\nu }_{2})+2|{E}_{F}|]}^{2}}{{[\hslash (\omega +i{\nu }_{2})-2|{E}_{F}|]}^{2}+{(2{k}_{B}T)}^{2}})\}\end{array}$$

where, *e* is the electronic charge, *k*_*B*_ is the Boltzmann constant, *T* is the temperature, *ħ* is the reduced Planck’s constant, *ω* is the angular frequency, *E*_*F*_ is the Fermi-level of graphene, and *ν*_1_ and *ν*_2_ are the scattering frequencies of the intraband and interband electrons respectively.

**Figure 2:**
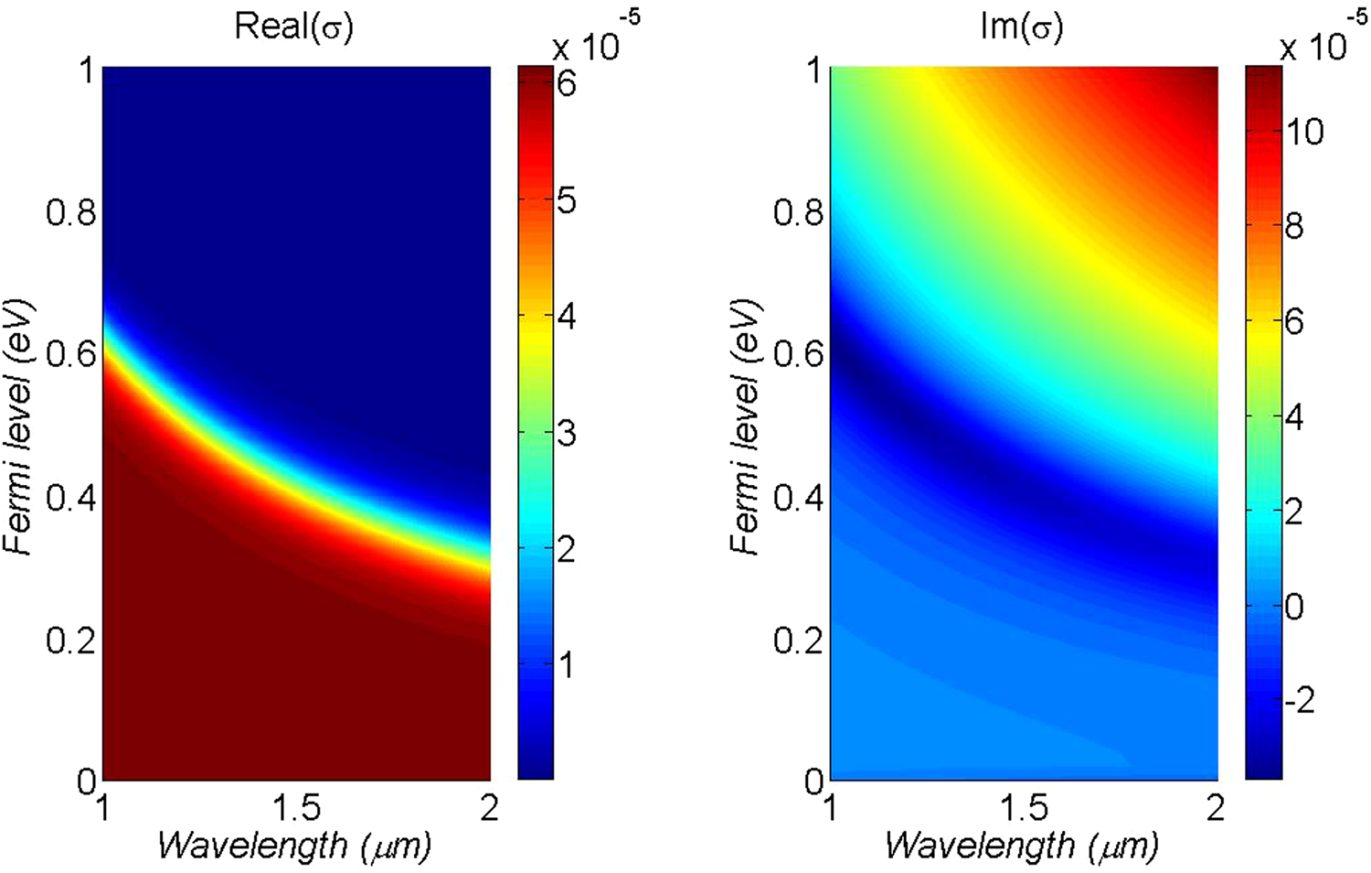
Graphene’s optical conductivity for wavelengths between 1 to 2 µm and Fermi levels between 0 to 1 eV.

Figure 2 shows the optical conductivity of graphene for wavelengths from 1 to 2 µm and *E*_*F*_ from 0 to 1 eV. For *E*_*F*_ below 0.2 eV, *σ*_*g*_ is relatively constant and predominantly real, hovering around the value of ${e}^{2}/4\hslash$, which translates to a material absorption coefficient of *α*_*g*_ = 10^6^ dB cm^−1^. The ultra-high material absorption and its stability over a broad spectrum will facilitate the design of compact and broadband devices.

Finally, the effective waveguide absorption can be assessed using

5 $$\alpha =4.34\times \frac{{\rm{Re}}({\sigma }_{g})/2}{{P}_{in}}{{\int }_{Wg}|{E}_{||}|}^{2}dx$$

where only the real part of *σ*_*g*_ is considered, with the square of in-plane electric-field magnitudes evaluated over the line integral along the width of the graphene layer, and *P*_*in*_ is the total input power^36^. All values can be obtained from mode solutions using the finite element method in COMSOL.

## Results and Analysis

### TE_0_ Filter

**Figure 3:**
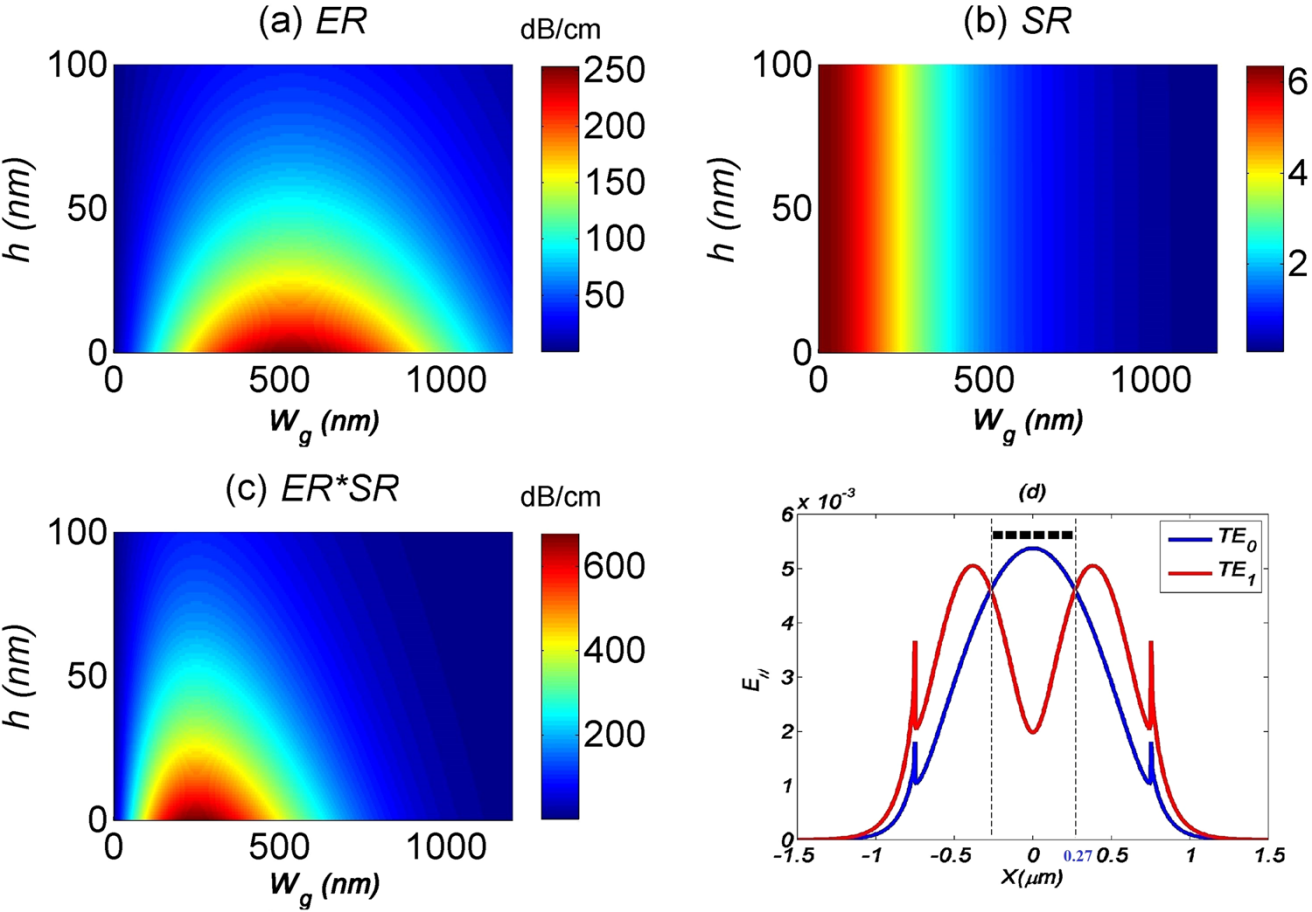
Performance parameters (**a**) ER, (**b**) SR and (**c**) ER-SR product for MF1 with respect to graphene’s width. (**d**) E_||_ intensity profile for both TE_0_ and TE_1_ modes across the GOS waveguide. Dashed lines indicate the coverage of the graphene layer.

In our first set of studies, we examine how *W*_*g*_ would affect the performance of the TE_0_ filter. The waveguide dimensions are fixed at *W* = 1500 nm and *H* = 200 nm. From Fig. 3(a), there is an optimum *W*_g_ that gives the highest *ER*. This is intuitively understood by looking at the electric-field profiles of the TE_0_ and TE_1_ modes as shown in Fig. 3(d), whereby the peak electric-field intensity of the TE_0_ mode is located at the center of the waveguide, while it is symmetrically displaced from the center for the TE_1_ mode. As a rule of thumb, to get the maximum *ER*, the graphene layer should spatially cover the waveguide up to the point where the electric-field intensities of the TE_0_ and TE_1_ modes are equal, as demarcated by the vertical grey lines in Fig. 3(d). This is confirmed by measuring the width of the demarcation in Fig. 3(d) to be *W*_*g*_ = 540 nm, which corresponds exactly to the maximum *ER* in Fig. 3(a). Meanwhile, *ER* will decrease with increasing *h*, as there is a decrease in modal overlap of both the TE_0_ and TE_1_ electric-field intensities with the graphene layer.

In Fig. 3(b), we see a linear decrease of *SR* with *W*_*g*_, which is obvious as the absorption of the TE_1_ mode is proportional to the area of the graphene layer. On the other hand, *SR* does not vary with *h*, due to the uniform decrease of both the modal overlaps of TE_0_ and TE_1_ electric-field intensities. Finally, the *ER-SR* product is maximum at *W*_*g*_ = 250 nm, as shown in Fig. 3(c). Overall, this shows that the graphene layer should adhere as closely as possible to the waveguide surface, while design of the *W*_*g*_ should take into consideration the tradeoff between device footprint and insertion losses.

For GOS waveguides with dimensions other than *W* = 1500 nm and *H* = 200 nm, *W*_*g*_ and *h* will affect the mode filter’s performance in the same style with Fig. 3. Maximum *ER-SR* product is dependent on the waveguide dimension *W* and *H*.

**Figure 4:**
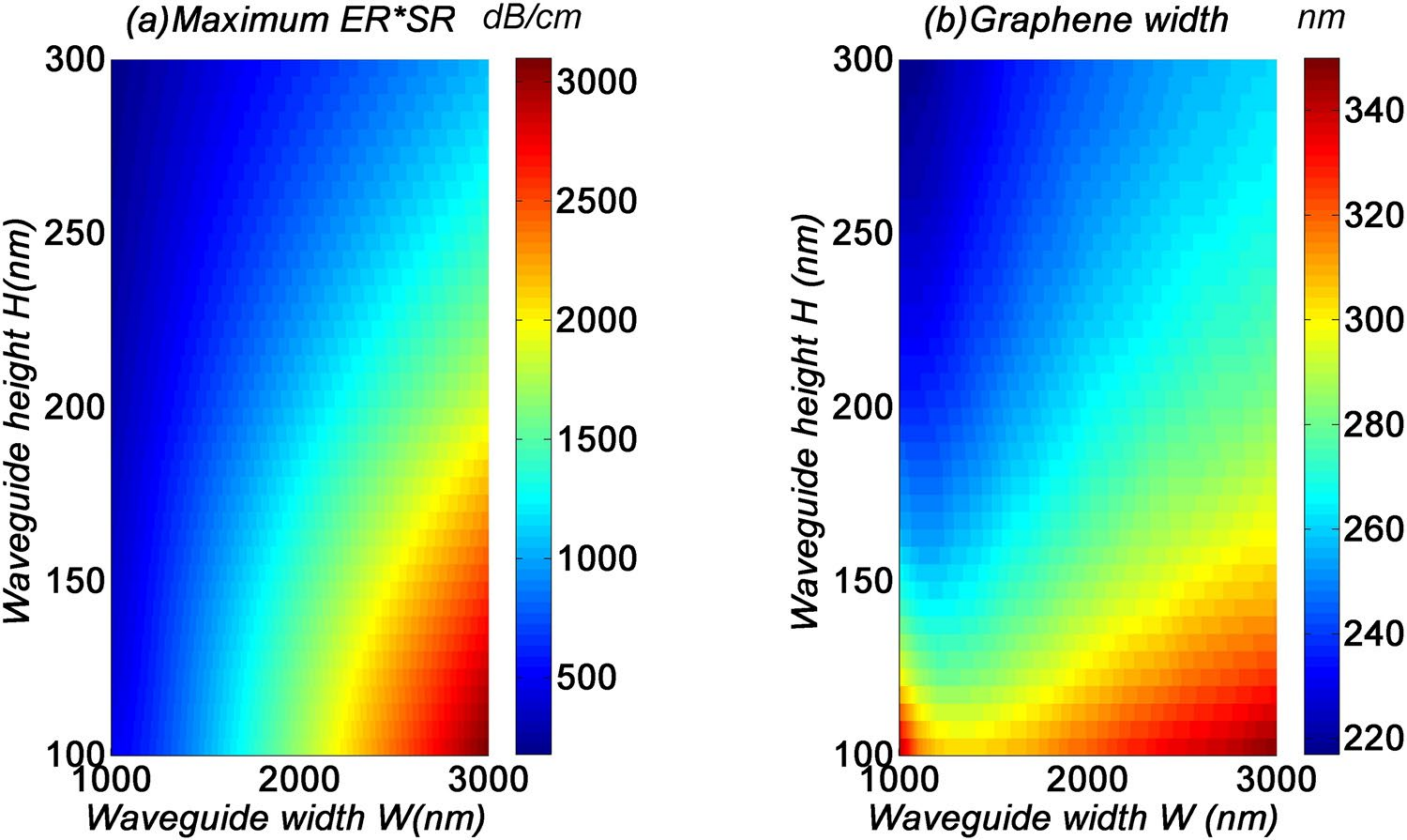
(**a**) Maximum ER-SR product, and (**b**) corresponding graphene’s width for MF1 with respect to the GOS waveguide dimensions W and H.

Finally, in Fig. 4 we look at how the maximum *ER-SR* product is affected by the waveguide dimensions. From Fig. 4(a), we see that in general, the waveguide width should be as large as possible, and the height as small as possible. Meanwhile, since the variation of the waveguide width would change the spatial location of the modes, the optimal *W*_*g*_ would thus scale accordingly, as shown in Fig. 4(b).

### TE_1_ Filter

**Figure 5:**
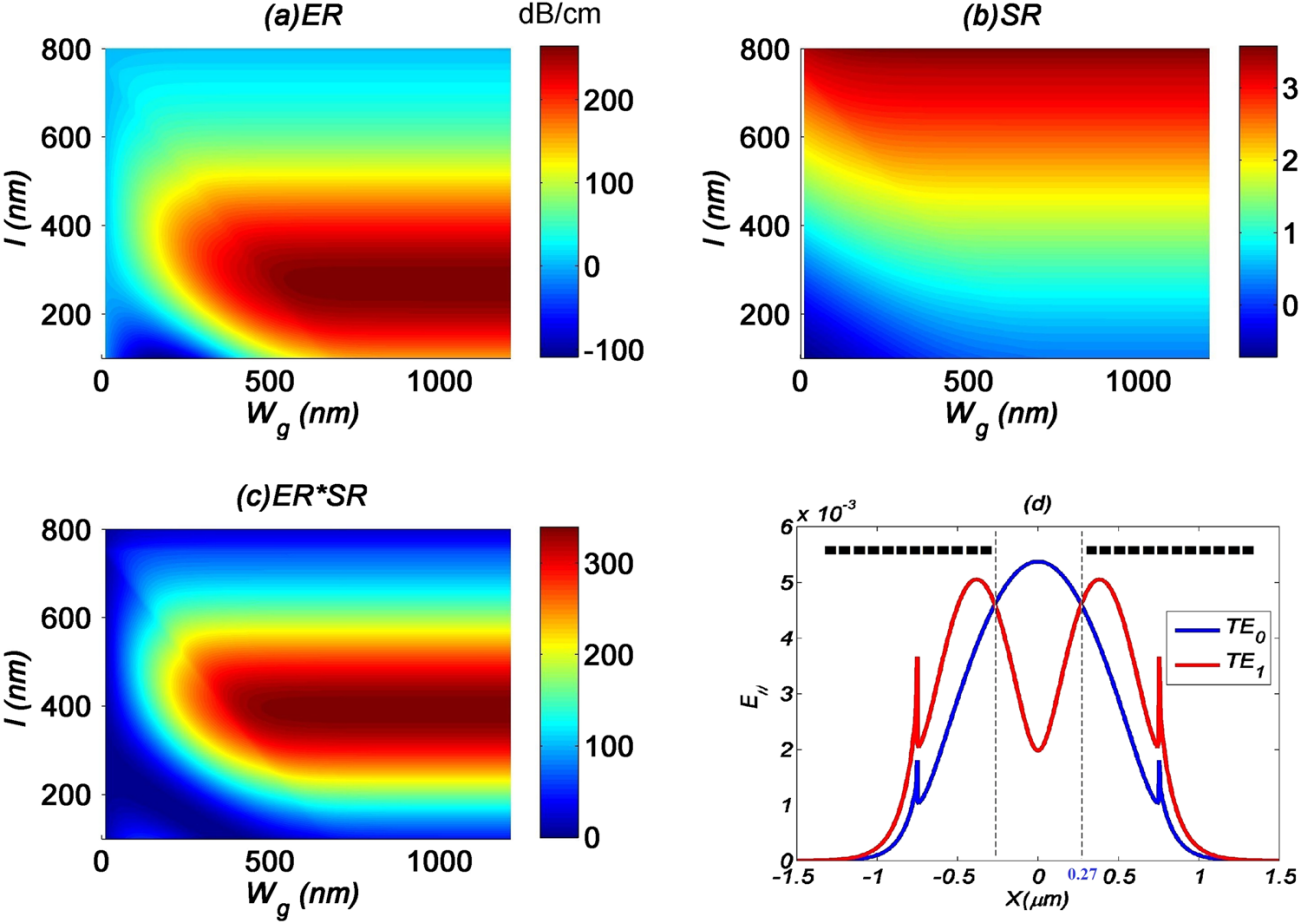
Performance parameters (**a**) ER, (**b**) SR and (**c**) ER-SR product for MF2 with respect to graphene width W_g_ and separation l. (**d**) E_||_ intensity profile for both TE_0_ and TE_1_ modes across the GOS waveguide. Dashed lines indicate the coverage of the graphene layer.

We conduct a similar analysis for the TE_1_ filter, with the same waveguide dimensions of *W* = 1500 nm and *H* = 200 nm, and the graphene strips are in direct contact with the waveguide surface. The usual performance parameters are shown in Fig. 5. From Fig. 5(a), it is observed that the maximum *ER* could be obtained by following an analogous rule of thumb: the displacement of the graphene strips from the waveguide center, *l*, should occur at the point where the electric-field intensities of the TE_0_ and TE_1_ modes are equal, as depicted in Fig. 5(d). This worked out to be 270 nm, which directly corresponds to the value found in Fig. 5(a). On the other hand, *W*_*g*_ variations are less critical as long as the strips are able to cover the entire waveguide surface. For example, when *l* = 270 nm, each strip should cover at least 480 nm to the waveguide edge, while when *l* = 200 nm, this width should be 550 nm.

Meanwhile, SR increases monotonically with *l*, and doesn’t vary with *W*_*g*_, as shown in Fig. 5(b). This is understood as when the graphene strips move away from the center, the TE_0_ mode experiences lower losses. Taken together, the *ER-SR* product has a maximum at *l* = 400 nm as seen in Fig. 5(c).

## Bandwidth analysis

**Figure 6:**
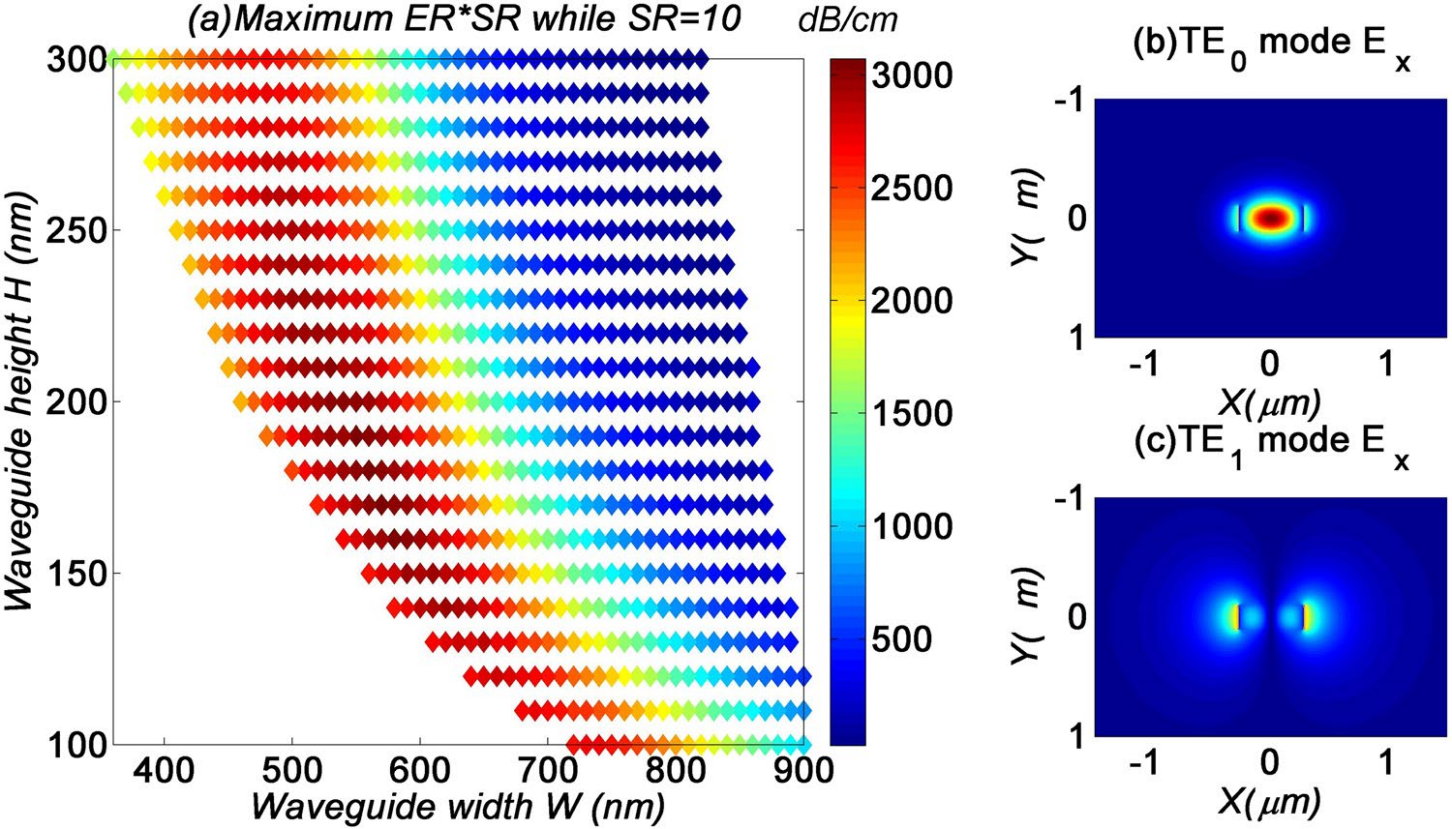
(**a**) Maximum ER-SR product MF2 with respect to the GOS waveguide dimensions W and H while SR = 10. TE_0_ (**b**) and TE_1_ (**c**) mode profile of the 560 × 200 nm waveguide.

In this section, we study the bandwidth performance of the GOS mode filters. Here, we use device parameters which are optimized to produce a signal contrast of 20 dB at the 1.55 µm wavelength. For the TE_0_ mode filter, as shown in Fig. 4(a), the device parameters are *W* = 3 µm, *H* = 100 nm, and *W*_*g*_ = 350 nm, which yield *ER* = 310 dB cm^−1^ and *SR* = 10. For better comparison of TE_0_ and TE_1_ mode filters, we choose the TE_1_ filter with the same *SR* = 10. While *SR* = 10, the optimized parameters with the maximum *ER-SR* product shown in Fig. 6(a) are *W* = 560 nm, *H* = 200 nm, and *l* = 365 nm, which yield *ER* = 307 dB cm^−1^. The modes profile of TE_1_ filter are drawn as Fig. 6(b,c). These two optimized structure are also used in the bandwidth and fabrication tolerance analysis.

**Figure 7:**
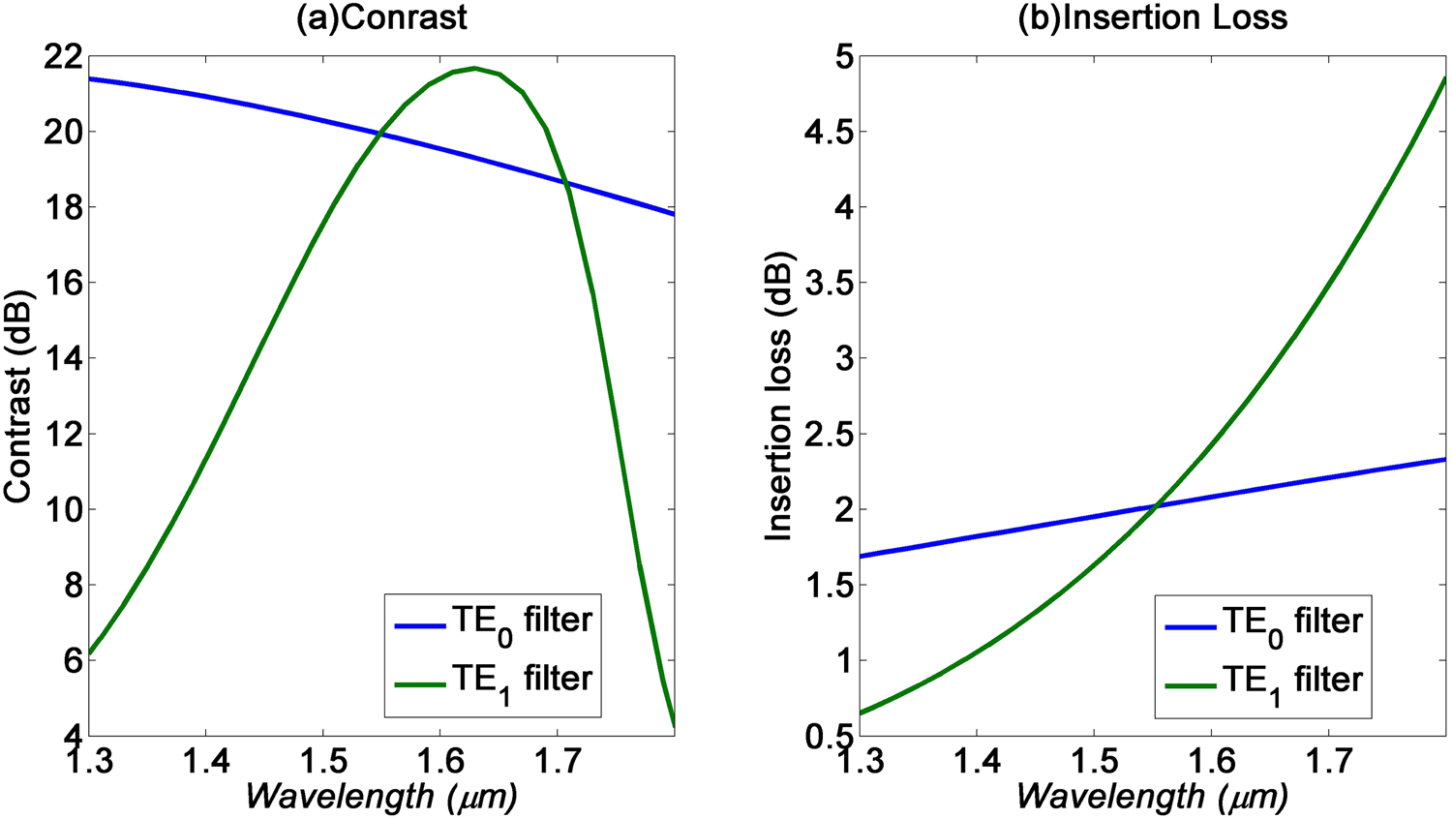
(**a**) Contrast and (**b**) insertion loss of the TE_0_ and TE_1_ filters respectively, for the spectral range from 1.3 to 1.8 µm.

Figure 7 shows the contrast and insertion loss of both mode filters for the spectral range from 1.3 to 1.8 µm. From Fig. 7(a), the TE_0_ filter has a broad 3 dB bandwidth of around 450 nm, while the TE_1_ filter has a narrower 3 dB bandwidth of 200 nm. For TE_1_ filter, the contrast is decreasing beyond 1.65 µm. While increasing the wavelength beyond 1.65 um, the TE_1_ mode electric field interacting with graphene layer is improved. However, the in-plane electric field is decreasing while the out-plane electric field is increasing. As a result, the TE_1_ mode absorption coefficient will decrease which will lead to the decrease in the contrast. Similarly, we see from Fig. 7(b) that the insertion losses for the TE_0_ filter is relatively constant at 2 dB, while that of the TE_1_ filter could spectrally vary from 0.5 to 5 dB. Therefore, while the absorption of graphene is relatively constant over a very wide spectral range, the absorption bandwidth is still limited by the spatial mode dispersion of the waveguide. This dispersion is more critical to the bandwidth performance of the TE_1_ filter, due to the fact that the graphene strips’ absorption is very sensitive to the spatial dispersion of the peak electric-fields of TE_1_ mode.

## Fabrication tolerance analysis

In a typical fabrication process of GOS mode filters, the graphene layer is first transferred to a pre-fabricated silicon waveguide, and then patterned into graphene strip(s) by lithography. Depending on the resolution of the lithographic equipment, the patterning of the graphene layer may induce fabrication errors to *W*_*g*_. In the electron beam lithography (EBL) process, the fabrication error is usually in the range of ± 20 nm. Another potential imperfection introduced into the fabrication process would be the alignment offset between the graphene strip and waveguide. The alignment offset in the EBL process is usually up to 40 nm. Here, we will study if these fabrication imperfections would affect the filtering performance, for the same device parameters listed in the previous section.

**Figure 8:**
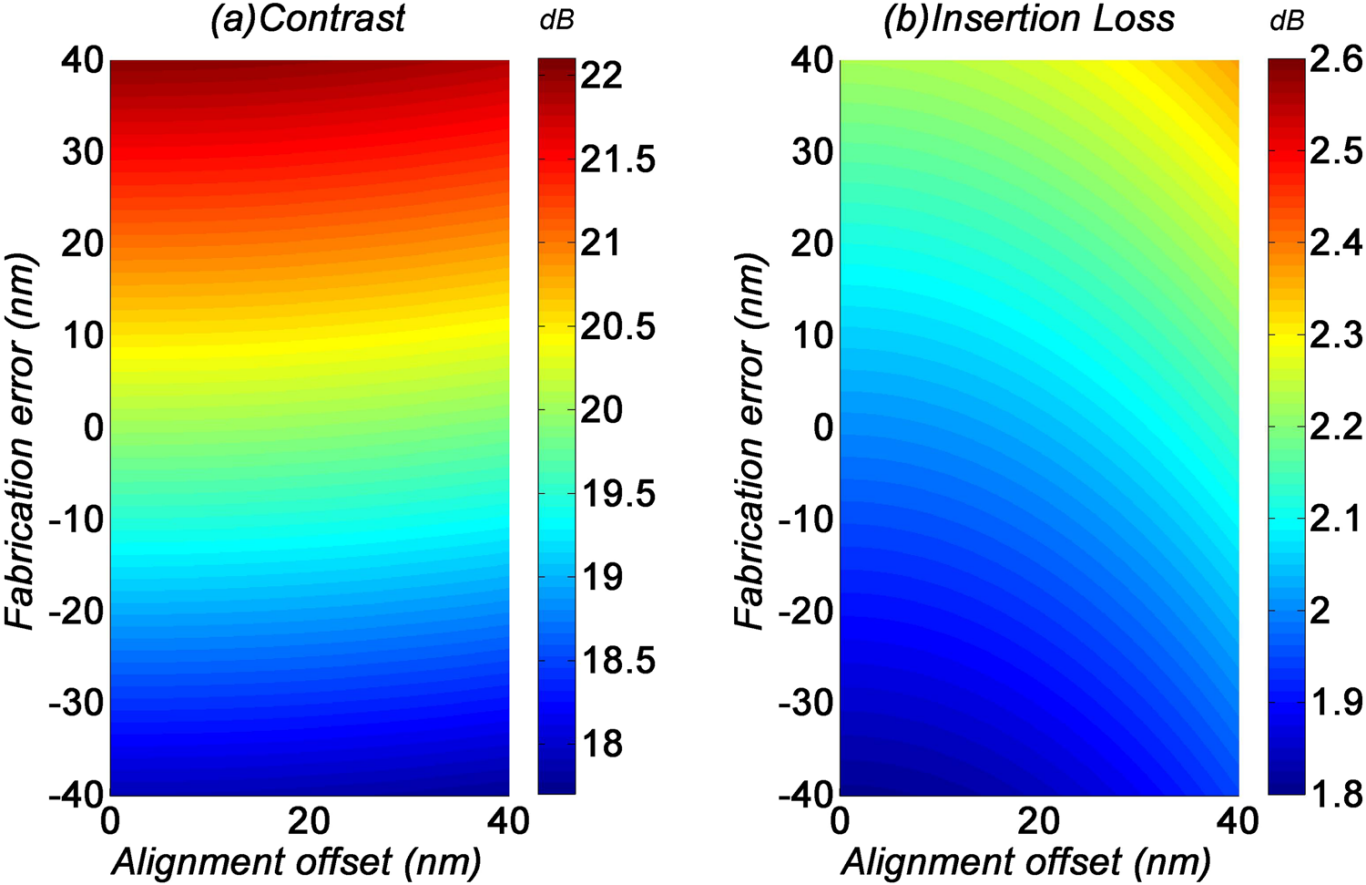
(**a**) Contrast and (**b**) insertion loss of the TE_0_ filter with fabrication imperfections.

The performance variations of the TE_0_ filter induced by fabrication imperfections is shown in Fig. 8. For this filter, the alignment offset does not significantly affect the contrast and insertion loss. On the other hand, the fabrication error of *W*_*g*_ induces a swing of ±2 dB to the contrast and ±0.5 dB to the insertion loss. The minimal impact to the device performance indicates that the TE_0_ filter has very good fabrication tolerance.

**Figure 9:**
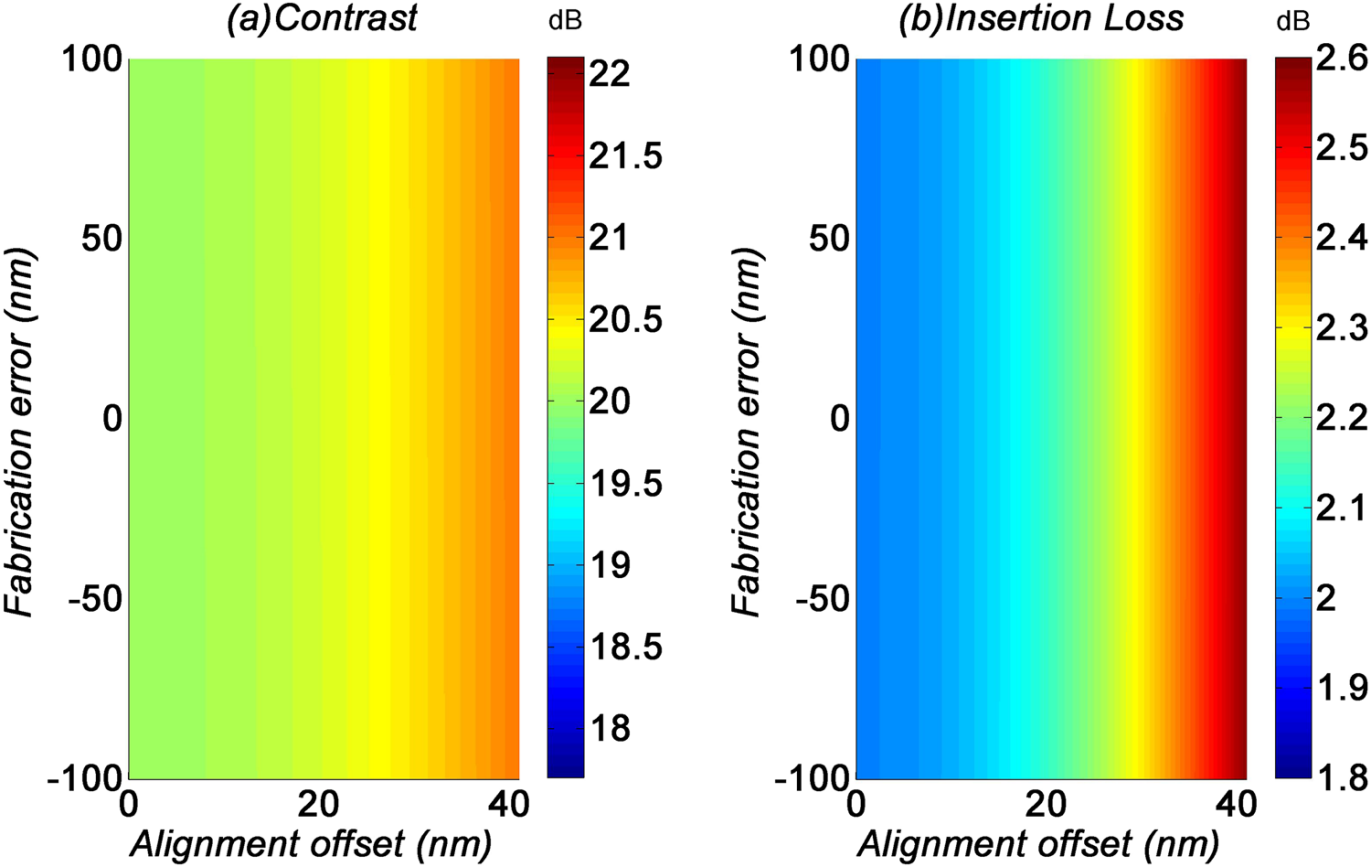
(**a**) Contrast and (**b**) insertion loss of the TE_1_ filter with fabrication imperfections.

Meanwhile, the performance variations of the TE_1_ filter induced by fabrication imperfections is shown in Fig. 9. For a large *W*_*g*_, the contrast and insertion loss are not affected by fabrication errors. The alignment offset of up to 40 nm would only induce a swing of ±0.5 dB to both the contrast and insertion loss. This shows that the TE_1_ filter has an equally good fabrication tolerance.

**Figure 10:**
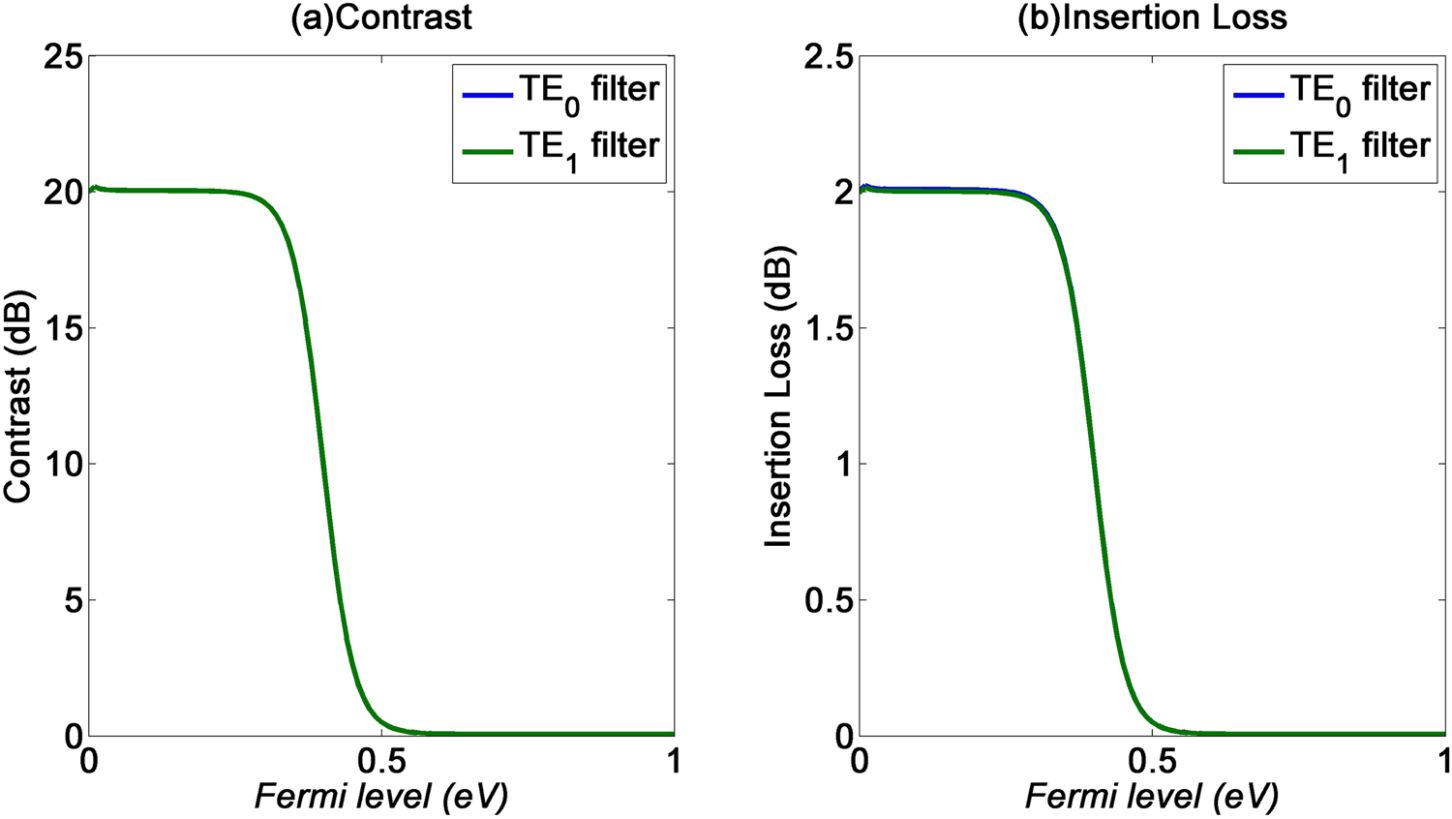
(**a**) Contrast and (**b**) insertion loss of the GOS mode filters with respect to graphene’s Fermi level.

Finally, we look at the effects of the Fermi level variation of the graphene layer, which is modified by dopants from the PMMA or other polymer used during the transfer process. From Fig. 10, the contrast and insertion losses are stable for low Fermi levels. However, as the Fermi level of graphene increases, its transition from insulator to metallic nature would degrade the absorption strength and thus lower both the contrast and insertion loss of the devices. It is thus important to keep the Fermi level low by removing excess dopants from the fabrication process.

## Conclusion

In summary, we have designed GOS waveguide mode filters to suppress the propagation of spurious waveguide modes, which function as auxiliary components to enhance the performance of existing MDM systems. There are stringent design requirements for MDM systems to avoid modal crosstalk, especially for waveguides designed for higher-order modes, which are influenced by the critical dimensions of the devices^3^. Integrating mode filters into the system may allow relaxing of the design and fabrication stringency, as the unwanted modes may be absorbed and removed from the signal channels. This would allow MDM designers to adopt higher spectral bandwidth devices, as the disadvantage of low coupling contrast in these devices are resolved via the waveguide mode filters. Our design of the GOS waveguide mode filters can achieve high selection and extinction ratios for a broadband spectrum around the telecommunications wavelength, hence providing low insertion losses and high signal contrasts for relatively small device footprints. They are also easier to fabricate and their performance has higher tolerance to fabrication imperfections compared to the current crop of resonant mode filters. GOS waveguide mode filters may continue to improve the performance of MDM systems to pave the way for their eventual market adoption.
